# Editorial: Mechanisms of Lymphocyte Exclusion in the Tumor Microenvironment

**DOI:** 10.3389/fimmu.2022.908612

**Published:** 2022-04-29

**Authors:** Rieneke van de Ven, Carlo B. Bifulco, Jason J. Luke, Sarah E. Church

**Affiliations:** ^1^Amsterdam UMC, location Vrije Universiteit Amsterdam, Department of Otolaryngology/Head and Neck Surgery, Amsterdam, Netherlands; ^2^ Cancer Center Amsterdam, Amsterdam, Netherlands; ^3^Amsterdam Institute for Infection and Immunity, Amsterdam, Netherlands; ^4^Providence Genomics and Earle A. Chiles Research Institute, Portland, OR, United States; ^5^Providence Cancer Institute, Portland, OR, United States; ^6^Department of Pathology, Providence Health and Services – Oregon, Portland, OR, United States; ^7^Division of Hematology/Oncology, University of Pittsburgh Medical Center (UPMC) Hillman Cancer Center, Pittsburgh, PA, United States; ^8^Cancer Immunotherapeutics Center, University of Pittsburgh Medical Center, Pittsburgh, PA, United States; ^9^NanoString Technologies, Inc., Seatlle, WA, United States

**Keywords:** tumor micreoenvironment (TME), lymphocyte exclusion, anti-tumor immune response, immune cell infiltration, immune suppression

Solid tumors which have an abundance of lymphocytes penetrating the tumor fields, so-called immune inflamed tumors, often have a prognostic benefit over non-immune inflamed tumors, which generally respond less to treatment. Non-inflamed tumors can be classified as immune deserts, with no lymphocytes present within the tumor microenvironment (TME), or immune excluded, where lymphocytes are unable to penetrate the tumor core from the stromal areas. This Research Topic includes 14 articles that explore the mechanisms that drive lymphocyte exclusion and provide insight how to attract the lymphocytes into the tumor nests.

Zhang et al. elaborately review the chemokines and cytokines that are needed to recruit, expand, differentiate and nurture T cells within the TME and those that promote T cell absence, exclusion, exhaustion and apoptosis. Pietrobon et al. provide an informative review on the conventional and next generation imaging technologies available to interrogate the TME as a means to better understand the mechanical, functional and dynamic barriers that underlie immune exclusion. In an original research article, Campisi et al. studied one of these barriers, using an *in vitro* microfluidics system to mimic a vascularized tumor environment. They show that tumor-derived 2’3’-cGAMP was able to, through STING, activate endothelial cells. This promoted production of lymphocyte-recruiting chemokines and adhesion molecules on the endothelium required for lymphocyte extravasation and recruitment. Several cancers, among which KRAS-LKB1 mutant lung cancers, are irresponsive to immune checkpoint treatment with anti-programmed death 1 (PD-1) agents. The authors show that loss of LKB1 activity impaired tumor 2’3’ cGAMP production, blocking T cell recruitment *via* the vasculature.

Not only the vasculature can prevent T cells from entering the TME, growing scientific evidence is pointing towards an obstructive role of suppressive myeloid cell populations within the TME. Asiry et al. extensively review the role of M2-type tumor-associated macrophages (TAMs) in regulating T cell trafficking into the tumor area and supporting the formation of so-called Tumor MicroEnvironment of Metastasis (TMEM) doorways, which aid in cancer cell dissemination and metastatic spread. These TMEM are characterized by the presence of tumor cells highly expressing the actin-regulatory protein Mammalian enabled (MENA), perivascular macrophages expressing the M2-linked tyrosine kinase receptor TIE2, and endothelial cells. *Via* a process that requires the interaction of a tumor cell with an M2-polarized TAM, the tumor cells acquire invasive features and are “streamed” towards the TMEM doorways, where they can metastasize. This concept is referred to as the “*dissemination trajectory*”. While T-cell directed immunotherapies may potentiate the attack of the bulk of the proliferating tumor, there is a possibility that cancer cells within a dissemination trajectory are shielded from T-cell mediated killing. Sticking with myeloid cells, Mehta et al., elegantly review macrophage biology and their role in immune suppression in breast cancer. Breast cancer cells secrete multiple factors that recruit monocytes and promote the skewing towards M2-like TAMs. Also in breast cancer, specific TIE2+ TAMs have been linked to promotion of angiogenesis and inhibition of T cell specific immune responses. In their review Mehta et al. discuss the current toolbox at hand, and those required in the future, to effectively target TAMs to promote T cell infiltration and functionality in breast cancer. A comprehensive review by the same group (Goldberg et al.) discusses the TME composition, novel therapeutic targets and potential combination therapies, focused at hormone receptor positive (HR+) breast cancer. Compared to HER2+ breast cancer and triple negative breast cancer (TNBC), HR+ tumors appear to have a lower level of TILs infiltrating into the tumor. These HR+ tumors are also characterized by high myeloid cell contents and low tumor HLA-I and PD-L1 expression and generally respond poorly to immune checkpoint inhibitor therapies. An interesting combination treatment, which was shown effective in preclinical *in vivo* models, and showed promising results in small cohorts of HR+ breast cancer patients in clinical studies, is the combination of a CDK4/6 inhibitor with PD-(L)1 inhibition.

Immune checkpoint therapy targeting PD-(L)1 and/or CTLA-4 barely induces responses in patients with advanced stage pancreatic ductal adeno carcinoma (PDAC). In a mini review, Vonderheide and Bear address that the chemokines secreted by PDAC cells are often driven by tumor-cell intrinsic factors such as KRAS mutations, and promote a myeloid-rich TME. In patients, targeting suppressive myeloid cells has not yet been very successful, and the authors discuss whether targeting tumor-intrinsic oncogenic pathways that drive chemokine production might be a more effective strategy to take forward. In their original research article, Raphael et al. identified PD-1 and TIGIT as the main immune checkpoint molecules related to poor survival in glioblastoma (GBM). Using a syngeneic GBM mouse model, they show that combination treatment of anti-TIGIT and anti-PD-1 inhibitors reduced tumor burden and improved survival. This coincided with increased CD8+ and CD4+ TIL frequencies in the tumor. Myeloid-derived suppressor cells (MDSCs) in GBM were found to express high levels of the ligands activating PD-1 and TIGIT. Combined blockade of TIGIT and PD-1 reduced polymorphonuclear (PMN)-MDSCs and increased the CD8 TIL/MDSC ratio in GBM tumors. Obviously myeloid cells are not the only type of cells within the TME that prevent the infiltration and functioning of effector T cells. In a detailed mini review, Scott et al. discuss the suppressive barriers that T regulatory cells (Treg) create at multiple levels at different sites: within the tumor by secreting suppressive cytokines and expressing multiple checkpoint molecules, altering the stromal compartment preventing effector T cells from reaching the tumor nests, deregulation of vasculature and obstructing dendritic cell (DC) activation or inducing DC apoptosis in tumor-draining lymph nodes (tdLNs), thereby preventing T cell priming and activation.

Liver cancer, including hepatocellular carcinoma (HCC), has seen unprecedented responses (30%) in the advanced disease setting since the introduction of immune checkpoint inhibitors. Lindblad et al. review the tumor-intrinsic mechanisms that may underlie immune cell exclusion, and unresponsiveness to checkpoint inhibition in the remaining 70% of non-responding patients. An important player in HCC, which has also been shown to regulate T cell exclusion in other cancer types, appears activation of the wnt/β-catenin (CTNNB1) pathway, which results in a paucity of cross-presenting DCs in the TME, preventing efficient downstream priming, activation and recruitment of effector T cell subsets. In a mini review, van Pul et al. emphasize the importance of DCs in shaping an effective anti-tumor T cell response and promoting T cell infiltration into tumors, focusing on the importance of tdLNs in generating the right T cells for tumor infiltration. Recent studies have elegantly shown that the effect of anti-PD-(L)1 immunotherapies relies on blockade of the PD-1/PD-L1 axis in the tdLNs in addition to the primary tumor site. This improves the priming of stem-cell like, progenitor exhausted T cells by LN-resident DCs, which can home to and infiltrate the TME and kill tumor cells upon anti-PD-1 therapy. In a comprehensive review, Blair et al. showcase the current literature on the relevance of antigen-specific interactions, T cell (re)circulation and T cell retention within the TME. Production of T-cell attracting chemokines by stromal cells, rather than by tumor cells, may contribute to a T cell excluded TME and increased recirculation of antigen-specific T cells, that may never reach the tumor cell nests to recognize and kill the tumor cells. While many, non-tumor specific T cells may be present within an immune inflamed TME, the ability of a TME to retain the antigen-specific T cells in close proximity to tumor cells, rather than allowing them to leave the tumor *via* lymphatic- or blood vessels may ultimately determine the effectiveness of an anti-tumor T cell response, as well as therapies that aim at reinvigorating such a response.

Since patients with T-cell excluded TMEs in general respond less well to the current immunotherapy strategies, these patient may benefit from combination treatments rather than monotherapy. In a mini review, Kacew and Sweis discuss the rationale for combining immune checkpoint inhibitors with agents inhibiting the fibroblast growth factor receptor-3 (FGFR3) in patients with urothelial bladder cancer. In patients irresponsive to checkpoint inhibitors, due to a non-immune inflamed TME, or patients with acquired resistance upon initial treatment, inhibition of FGFR3 may overcome T cell exclusion. An ideal way to stratify patients for immunotherapy would be by using a blood-based assay, rather than requiring a tumor biopsy to assess protein or gene expression. In an original research article, Younis et al. show for head and neck squamous cell carcinoma, that patients with high levels of the soluble glycoprotein semaphorin 4D (SEMA4D) in plasma displayed an immune excluded or desert TME type whereas patients with low soluble SEMA4D levels often displayed an immune inflamed phenotype, linked with high interferon immune signatures. Whether this can be used as a predictive biomarker of response to checkpoint inhibitors, requires further assessment.

We hope the readers of this Research Topic, focusing on mechanisms of lymphocyte exclusion from the tumor microenvironment, will appreciate the comprehensive overview of the different barriers T cells encounter in order to reach the tumor nests. With this overview, we would like to encourage the field to explore the suggestions provided by the contributing authors, working towards more means to overcome immune exclusion.

## Author Contributions

SC initiated the Research Topic. All authors edited the Research topic. RV wrote the editorial. All authors contributed to the editorial article and approved the submitted version.

## Conflict of Interest

RV receives research support from Genmab BV. CBis in the Scientific Advisory Boards of: PrimeVax (stock), BioAI (stock) and Lunaphore (no stock). JL discloses the following: member within Data and Science Monitoring Boards: Abbvie, Immutep, Evaxion; Scientific Advisory Board: (no stock) 7 Hills, Bright Peak, Exo, Fstar, Inzen, RefleXion, Xilio (stock), Actym, Alphamab Oncology, Arch Oncology, Kanaph, Mavu, NeoTx, Onc.AI, OncoNano, Pyxis, STipe, Tempest; Consultancy with compensation: Abbvie, Alnylam, Bayer, Bristol-Myers Squibb, Castle, Checkmate, Codiak, Crown, Day One, Duke St, EMD Serono, Endeavor, Flame, Genentech, Gilead, HotSpot, Kadmon, Janssen, Ikena, Immunocore, Incyte, Macrogenics, Merck, Mersana, Nektar, Novartis, Partner, Pfizer, Regeneron, Servier, STINGthera, Synlogic, Synthekine. Research Support: (all to institution for clinical trials unless noted) AbbVie, Astellas, Astrazeneca, Bristol-Myers Squibb (IIT & industry), Corvus, Day One, EMD Serono, Fstar, Genmab, Ikena, Immatics, Incyte, Kadmon, KAHR, Macrogenics, Merck, Moderna, Nektar, Next Cure, Numab, Palleon, Pfizer (IIT & industry) Replimmune, Rubius, Servier (IIT), Scholar Rock, Synlogic, Takeda, Trishula, Tizona, Xencor; Patents: (both provisional) Serial #15/612,657 (Cancer Immunotherapy), PCT/US18/36052 (Microbiome Biomarkers for Anti-PD-1/PD-L1 Responsiveness: Diagnostic, Prognostic and Therapeutic Uses Thereof). SC is a stockholder and employee of NanoString Technologies, Inc.

## Publisher’s Note

All claims expressed in this article are solely those of the authors and do not necessarily represent those of their affiliated organizations, or those of the publisher, the editors and the reviewers. Any product that may be evaluated in this article, or claim that may be made by its manufacturer, is not guaranteed or endorsed by the publisher.

